# Brief intervention, lasting impact: One-year outcomes of the Bergen 4-day treatment for OCD in Germany

**DOI:** 10.1371/journal.pone.0350615

**Published:** 2026-06-25

**Authors:** Lena Jelinek, Amir H. Yassari, Saskia Pampuch, Franziska Miegel, Josephine Schultz, Bjarne Hansen, Kristen Hagen, Jürgen Gallinat, Jakob Scheunemann

**Affiliations:** 1 Department of Psychotherapy and Psychiatry, University Medical Center Hamburg-Eppendorf, Hamburg, Germany; 2 Center for Brain Plasticity, Haukeland University Hospital, Bergen, Norway; 3 Møre and Romsdal Hospital Trust, Division of Psychiatry, Molde, Norway; Southwest University, CHINA

## Abstract

**Background:**

Obsessive–compulsive disorder (OCD) is a prevalent and highly disabling mental disorder. Worldwide, it is associated with substantial individual suffering and long-term societal costs. Although exposure and response prevention (ERP) is the recommended first-line treatment, access to effective care remains limited for reasons such as long treatment duration. These barriers contribute to persistent global treatment gaps. The Bergen 4-Day Treatment (B4DT) is a brief, concentrated form of ERP (cERP) designed to improve treatment efficiency and access. Although initial outcomes are promising, long-term data from countries other than Norway are limited. This study evaluated the 12-month outcomes of the B4DT in a German day-patient treatment setting, exploring its sustained effectiveness and feasibility outside its original context.

**Methods:**

Fifty-eight adults with OCD received B4DT in an uncontrolled study and were reassessed 12 months after treatment. The primary outcome was OCD severity, assessed with the Yale-Brown Obsessive Compulsive Scale (Y-BOCS). Secondary outcomes included depressive symptoms, global functioning, self-efficacy, experiential avoidance, self-esteem, quality of life, and mental health service utilization.

**Results:**

Large reductions in OCD symptom severity (Y-BOCS) and significant improvements across most secondary outcomes were sustained at the 12-month follow-up. One year after treatment, 69% of participants met criteria for treatment response, 51% had achieved remission, and 78% showed reliable clinical improvement. No clinically relevant symptom deterioration was observed, and treatment acceptability and follow-up retention were high. Importantly, only a small proportion of patients (*n* = 4; 7%) required inpatient treatment during the follow-up period.

**Conclusion:**

These findings demonstrate that brief cERP can achieve durable clinical benefits for patients with OCD when implemented outside its original context. From a global mental health and health policy perspective, the combination of short treatment duration, sustained effectiveness, and low subsequent use of inpatient services suggests that cERP represents a scalable and resource-efficient strategy to expand access to evidence-based OCD treatment across diverse health care systems.

## Introduction

Obsessive–compulsive disorder (OCD) is a chronic and highly disabling mental disorder affecting 1–3% of the global population [1,2]. It is associated with marked impairment in quality of life, social participation, and occupational functioning [3–5], and exerts a substantial burden not only on affected individuals but also on families and caregivers [6–8]. From a public health perspective, OCD is linked to considerable long-term societal and economic costs, driven by its chronic symptoms, repeated treatment episodes, and high utilization of specialized services [9–11].

Exposure and response prevention (ERP) has demonstrated superiority over waitlist, placebo, and non-specific psychological interventions and is the recommended first-line treatment for OCD in treatment guidelines [12,13]. In ERP therapists guide their patients to gradually expose themselves to feared thoughts or situations without performing compulsions, ultimately reducing the anxiety triggered by the obsessions [14]. However, despite its strong efficacy under controlled conditions, ERP remains insufficiently available in routine care worldwide [15]. In real-world settings, only about half of patients achieve clinically meaningful improvement [16], long-term effect sizes tend to diminish over time [17], and dropout rates and patient refusal to initiate ERP remain significant challenges [18,19]. These limitations stem not only from patient-related factors but also arise from structural constraints, including lengthy treatment durations, high demands on therapist time, limited specialist availability, and challenges in implementing concentrated exposure-based interventions in different settings. As a consequence, many patients remain untreated, exacerbating treatment gaps and prolonging the course of illness.

In response to these challenges, clinicians in Norway developed the Bergen 4-Day Treatment (B4DT) as a brief, concentrated form of ERP (cERP) aimed at improving treatment efficiency and access. The treatment emphasizes rapid engagement, teaching patients how to perform exposures for optimal and rapid effect while also equipping them with strategies for implementing these skills in their daily lives to ensure both immediate and sustained therapeutic outcomes. Evidence from predominantly uncontrolled studies conducted in Scandinavia indicates high short-term response and remission rates [Finland: 20, Norway: 18, 21, 22, Iceland: 23, 24], with one randomized controlled trial reporting response rates exceeding 90% [25].

Outside Scandinavia, the evidence is limited. In Germany, the first implementation of cERP modeled on B4DT showed large short-term effects compared to matched historical inpatient controls as well as high acceptability in routine care [26]. In the subsequent continuation of the trial, recruitment expanded to a total sample of *N* = 58 patients treated with B4DT. Miegel et al. [27] reported that in this sample 63% met criteria for responder and 54% achieved remission at post-assessment. At the 3-month follow-up, 61% remained responders, with 54% still meeting remission criteria. One other study outside of Scandinavia has been published, with data from the United States [28]; it reports positive outcome effects up to 6 months after treatment and high acceptability ratings. To date, however, no data are available on 12-month outcomes of B4DT outside Norway. From a global mental health and health policy perspective, such long-term data are essential to evaluate whether brief, intensive interventions can produce durable benefits and reduce ongoing reliance on resource-intensive care. Currently, long-term follow-up data, which indicate sustained remission over periods of up to four years, are only available for Norway [18,21].

Despite encouraging long-term outcomes reported in Norwegian studies, an important health systems question remains insufficiently addressed. While concerns about post-treatment service utilization are particularly relevant for nonrandomized designs, follow-up mental health care is rarely reported systematically across study types. As a result, the extent to which sustained improvements following B4DT reflect the durability of the initial intervention versus continued access to specialized services, including cost-intensive inpatient or day-patient treatment, remains unclear. From a health policy and global mental health perspective, systematically assessing service utilization during follow-up is therefore essential for evaluating the real-world sustainability and scalability of brief concentrated treatments.

Against this background, the present study not only examined the durability of clinical outcomes following B4DT but also systematically assessed mental health service utilization during the 12-month follow-up period, with a particular focus on inpatient and day-patient treatment.

### Aim of the present study

The present study extends previous findings by evaluating the 12-month outcomes of brief, concentrated exposure and response prevention (B4DT) in a German day-patient treatment setting. The primary aim was to assess the durability of treatment effects on clinician-rated OCD symptom severity (Yale-Brown Obsessive-Compulsive Scale, Y-BOCS [29]). Secondary aims included examining long-term outcomes in self-rated OCD symptoms, quality of life, depressive symptoms, self-esteem, experiential avoidance, self-efficacy, and psychosocial functioning**.** In addition, we systematically assessed mental health service utilization during the 12-month follow-up period, with a particular focus on the use of inpatient and day-patient treatment. By providing the first 12-month follow-up data on B4DT outside Norway, this study seeks to inform clinical practice and health policy discussions on scalable, resource-efficient strategies to improve access to evidence-based OCD treatment and to evaluate whether B4DT may reduce the need for subsequent resource-intensive inpatient care in mental health care systems.

## Methods

### Design and procedure

As we have described previously [26,27], participants were recruited at the University Medical Center Hamburg-Eppendorf, Germany, between 5 September 2022 and 13 May 2024. All participants were enrolled and assessed at baseline during this period. Inclusion criteria required participants to be between 18 and 75 years of age, meet diagnostic criteria for obsessive-compulsive disorder (OCD) according to DSM-5 criteria as determined by the Mini International Neuropsychiatric Interview [30], and have no history of psychotic disorders, mania, or substance dependence and have no acute suicidality. Participants were required to be either free of psychotropic medication or on a stable medication regimen for at least six weeks prior to treatment initiation.

**Fig 1:**
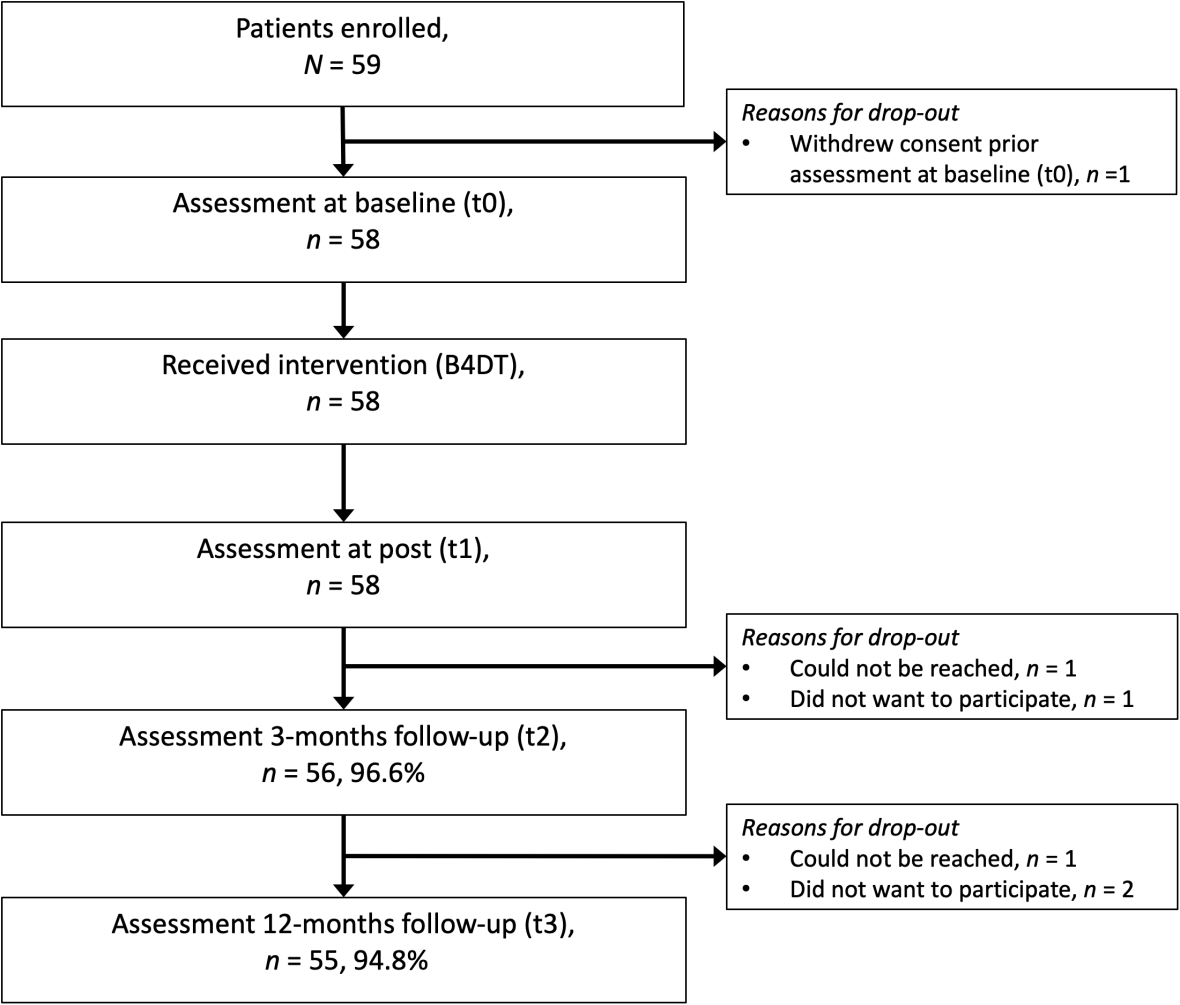
Flowchart.

As can be seen in Fig 1, a total of 59 patients were enrolled. Because the study was conducted in a routine clinical setting, the total number of patients assessed for eligibility was not systematically documented. One participant initiated treatment after a delay of three months and was included in the study, whereas one participant withdrew prior to assessment at baseline and treatment initiation. Accordingly, the final analytical sample consisted of 58 patients who were assessed at baseline (t0) and also received the intervention. Follow-up assessments were conducted at 3 and 12 months post-treatment, with the final follow-up completed on 1 July 2025.

OCD symptom severity was assessed using the Y-BOCS at four time points: prior to treatment (baseline, T0), two weeks after T0 (post-treatment, T1), three-month follow-up (T2), and 12-month follow-up (T3). All participants underwent cERP. The trial received approval from the local ethics committee (# LPEK-0512) and was preregistered with the German Clinical Trials Register (#DRKS00030022). All procedures complied with the Declaration of Helsinki, ensuring ethical conduct and protection of the participants. Written informed consent was obtained from all participants to participate in the study.

#### Brief concentrated therapy for obsessive-compulsive disorder.

Treatment was based on the B4DT protocol developed in Norway, a brief cERP for individuals with OCD. All therapists were trained and supervised by the Norwegian developers of B4DT. The core element of B4DT involves four consecutive days of treatment. Throughout this period, patients receive a blend of individual and group-based therapeutic support delivered by a multidisciplinary team (psychologists, psychiatrists, psychiatric nurses), with patients paired 1:1 with therapists. To help maintain treatment gains, a booster session of around 50 minutes was offered to all participants 3 months after the four treatment days, consistent with prior B4DT studies. Treatment followed a standardized protocol, and supervision was provided throughout the study period to ensure consistency with the B4DT model. For detailed descriptions of the treatment structure and therapeutic approach, see [31,32], which provide comprehensive accounts of the B4DT methodology and implementation.

#### Measures.

In line with the initial publication of the pilot results [26], we assessed OCD severity as the primary outcome using the Y-BOCS, with excellent interrater reliability. In this study, all Y-BOCS assessments were conducted by trained raters who were independent of the therapeutic process to ensure objectivity.

Secondary outcomes included assessment of self-reported OCD symptoms with the Obsessive-Compulsive Inventory-Revised [33, OCI-R, 34], depressive symptoms with the Patient Health Questionnaire-9 (PHQ-9; [35,36]), quality of life using the global item of the World Health Organization Quality of Life Questionnaire (WHOQOL-BREF; [37,38]), self-esteem with the Rosenberg Self-Esteem Scale (RSE; [39]), experiential avoidance with the Brief Experiential Avoidance Questionnaire (BEAQ; [40,41]), and self-efficacy with the General Self-Efficacy Scale (GSES; [42]), all of which demonstrated good to excellent internal consistency in the present sample and were filled out digitally. Additionally, psychosocial functioning was evaluated using the Global Assessment of Functioning (GAF; [43]).

The Client Satisfaction Questionnaire–8 (CSQ-8; [44]; German: ZUF-8; [45]) was again administered at the 12-month follow-up (T3) to measure the patients’ subjective appraisal of the B4DT.

To assess health care utilization during the 12-month follow-up period, we used a brief self-report questionnaire. Participants reported their number of days of sick leave in the past 12 months, current and past psychotropic medication use, and utilization of psychological treatment since completion of the B4DT. Information on initiation, termination, or change of psychotherapy, as well as the number of psychotherapy sessions, was recorded. In addition, participants indicated whether they had received inpatient or day-patient psychiatric treatment during the follow-up period.

#### Statistical analyses.

Changes in primary and secondary outcomes were analyzed using repeated measure analyses of variance (ANOVAs) with time as the within-subject factor and participant as the repeated factor. Paired *t*-test were used for comparisons between specific time points (e.g., post-treatment vs. follow-up). For treatment acceptability, descriptive data analyses of the CSQ-8 as well as dropout analyses were performed. Clinically relevant symptom deterioration and improvement were calculated using the reliable change index (RCI) as described by Jacobson and Truax [46]. Treatment response was defined as a reduction of at least 35% in the total Y-BOCS score, while remission was indicated by a score of 12 or below [47].

To explore predictors of long term treatment response and remission, two regression analyses were performed: first, a linear regression analysis predicting change in the primary outcome from baseline to 12-month follow-up assessment, and second, a logistic regression predicting remission status (in remission, not in remission). In both analyses demographic and clinical variables (participants‘ age, gender, highest education, early or late onset, presence of comorbidity), baseline severity of OCD (Y-BOCS), depressive symptoms (PHQ-9), and content of OCD (contamination-, checking-, taboo-related) were included as covariates.

No formal adjustment for multiple testing was applied; given the exploratory nature of the analyses and the focus on estimating long-term outcomes across multiple measures in an extension of a feasibility study, findings require replication.

We report effect sizes using partial eta squared for ANOVAs, referring to η_p_^2^ ≈ .01 as small, η_p_^2^ ≈ .06 as medium, and η_p_^2^ ≈ .14 as large effect sizes, based on Kinnear and Gray [48]. For the *t*-test, we report Cohen’s *d*, following the parameters of Cohen [49] for small (*d* ≈ 0.2), medium (*d* ≈ 0.5), and large (*d* ≈ 0.8) effect sizes. For health care utilization, all variables were analyzed descriptively using means, standard deviations, frequencies, and percentages. Analyses were performed using IBM SPSS Statistics, version 31.

#### Power calculation.

The sample size was not based on an a priori power calculation but reflects the cohort of patients included in a prior feasibility study [27], with the present analyses representing an extended follow-up. Accordingly, analyses were not designed to detect predefined effect sizes but to estimate the magnitude and stability of treatment effects over time.

## Results

### Participants

See Table 1 for demographics, treatment-related data, and diagnoses of the total sample. Of the 58 patients assessed at baseline, 56 and 55 could be reached and assessed with the Y-BOCS at the 3-month (T2) and 12-month (T3) follow-ups, respectively (retention rate: 96.6% and 94.8%). At baseline, patients’ Y-BOCS total scores ranged between 18 and 36 points (*M* = 25.38, *SD* = 3.53), with OCD content relevant to contamination (*n* = 29, 50%), checking (*n* = 34, 58.6%), and taboo themes (*n* = 31, 53.4%), according to the Y-BOCS checklist.

**Table 1:** Demographic, Symptom, and Treatment Data: Mean (*M*), Standard Deviation (*SD*), Frequency (*n*), and Percentage (%) of the Total Sample (*N* = 58).

	*M* or *n*	*SD* or %
Age (years)	34.76	12.50
Gender (male/female/diverse)	23/35/0	39.7/60.3/0
Highest education		
Secondary school diploma	5	8.6
Upper secondary diploma^a^	1	1.7
University entrance qualification (= A-levels)	19	32.8
Bachelor’s degree	10	17.2
Master’s degree	23	39.7
Age at onset (early [< 16 years] / late [≥ 16 years]), *N* = 57	27/30	47.4/52.6
Age at first psychiatric/psychological contact	24.95	9.17
Number of inpatient/day-patient stays	1.14	2.70
Currently in psychological treatment, *N* = 45	37	63.8
Comorbid Diagnoses		
Lifetime depressive disorder, *N* = 57	40	70.2
Panic disorder, *N* = 56	4	7.1
Social phobia	5	8.6
Generalized anxiety disorder	3	5.2
Agoraphobia, *N* = 56	3	5.4
Posttraumatic stress disorder, *N* = 56	1	1.8
Alcohol use disorder	4	6.9
Psychotic disorder	0	0
Anorexia, *N* = 56	1	1.8
Bulimia	1	1.7

Note. Y-BOCS = Yale-Brown Obsessive Compulsive Scale. ^a^ qualification for universities of applied sciences.

Information on other pharmacological or psychological treatments between post and 12-month follow-up assessment as well as days of sick leave is displayed in Table 2.

**Table 2:** Treatment Data for the 12-Month Follow-Up Interval: Mean (*M*), Standard Deviation (*SD*), Frequency (*n*), and Percentage (%).

	*M* or *n*	*SD* or %	*range*
Days of sick leave in the past 12 months, *N* = 50	25.02	72.90	0–365 days
– Number of patients with 0 days of sick leave	28	48%	
– Number of patients with 1–14 days of sick leave	9	18%	
Pharmacological treatment – No psychotropic treatment	29	50	
– Stable psychotropic treatment (no change within the past 12 months)	11	19	
– Termination of psychotropic treatment in the past 12 months, *N* = 54	4	6.9	
– Change in pharmacological treatment since end of B4DT, *N* = 54	4	6.9	
Number of sessions of psychotherapy after B4DT, *N* = 53	11.17	11.95	0-40
Number of patients receiving inpatient/day-patient treatment after B4DT, *N* = 53	4	6.9%	

#### Treatment effects.

As shown in Table 3, patients demonstrated significant improvement from pre- to post-treatment, and it was sustained over 3 and 12 months. Large effect sizes were observed for the primary outcome (Y-BOCS) and most secondary measures (OCI-R, GAF, GSES, BEAQ). Improvements in depression (PHQ-9), self-esteem (RSE), and quality of life were small to moderate. Overall, treatment effects remained stable one year after the intervention.

**Table 3:** Means (Standard Deviations), Paired *t*-Tests, and ANOVA Group Comparisons of Primary and Secondary Outcomes.

	*M (SD)*				*t-test*			*ANOVA*
	Baseline (T0), *N* = 58	Post (T1), *n* = 58	3-month FU (T2), *n* = 56	12-month FU (T3), *n* = 55	T0 vs. T1	T0 vs. T2	T0 vs. T3	
Y-BOCS total	25.38 (3.53)	14.51 (5.79)	14.11 (7.79)	13.87 (7.90)	*t*(57) = 15.418, *p*< .001, *d* = 2.024, CI_95%_ [1.57,2.47]	*t*(55) = 12.747, *p*<.001, *d* = 1.703, CI_95%_ [1.29,2.11]	*t*(54) = 12.113, *p*<.001, *d* = 1.633, CI_95%_ [1.23,2.04]	*F*(3, 159) = 86.905, *p*<.001, η²_*p*_ = .621
Y-BOCS Obsession	12.51 (2.21)	7.74 (2.94)	7.21 (3.62)	6.55 (3.99)	*t*(57) = 12.559, *p*<.001, *d* = 1.65, CI_95%_ [1.25,2.04]	*t*(55) = 10.50, *p* < .001, *d* = 1.40, CI_95%_ [1.03,1.77]	*t*(54) = 12.413, *p*<.001, *d* = 1.674, CI_95%_ [1.26,2.05]	*F*(3, 159) = 66.153, *p*<.001, η²_*p*_ = .555
Y-BOCS Compulsions	12.86 (1.80)	6.78 (3.22)	6.89 (3.64)	7.33 (4.26)	*t*(57) = 15.279, *p*<.001, *d* = 2.01, CI_95%_ [1.56,2.45]	*t*(55) = 12.051, *p*<.001, *d* = 1.61, CI_95%_ [1.21,2.01]	*t*(54) = 9.924, *p*<.001, *d* = 1.338, CI_95%_ [0.97,1.70]	*F*(3, 159) = 74.788, *p*<.001, η²_*p*_ = .585
GAF	64.26 (11.95)	74.77 (11.72), *N* = 57	79.36 (11.27)	77.67 (13.77)	*t*(56) = 8.811, *p*<.001, *d* = 1.151, CI_95%_ [1.50,0.83]	*t*(55) = 10.636, *p*<.001, *d* = 1.421, CI_95%_ [1.79,1.03]	*t*(54) = 6.769, *p*<.001, *d* = 0.921, CI_95%_ [1.24,0.60]	*F*(3, 153) = 39.515, *p*<.001, η²_*p*_ = .437
WHOQOL-BREF	3.00 (0.79)	3.70 (0.74), *N* = 56	3.52 (0.84), *N* = 54	3.54 (0.85), *N* = 52	*t*(55) =6.456, *p*<.001, *d* = 0.863, CI_95%_ [1.17,0.55]	*t*(53) =4.696, *p*<.001, *d* = 0.639, CI_95%_ [0.93,0.34]	*t*(51) =3.612, *p*<.001, *d* = 0.501, CI_95%_ [0.79,0.21]	*F*(3, 150) = 9.992, *p* = .003, η²_*p*_ = .167
PHQ-9	9.04 (5.48)	5.67 (5.12), *N* = 57	5.89 (4.41), *N* = 55	6.56 (5.80), *N* = 52	*t*(56) = 5.151, *p*<.001, *d* = 0.682, CI_95%_ [0.39,0.97]	*t*(54) = 6.163, *p*<.001, *d* = 0.831, CI_95%_ [0.51,1.14]	*t*(51) = 2.859, *p* = .003, *d* = 0.395, CI_95%_ [0.11,0.68]	*F*(3, 150) = 8.349, *p*<.001, η²_*p*_ = .143
OCI-R	25.39 (9.08)	12.035 (6.57), *N* = 57	13.38 (7.12), *N* = 55	15.57 (9.3), *N* = 53	*t*(56) = 10.275, *p*<.001, *d* = 1.361, CI_95%_ [1.00,1.72]	*t*(54) = 9.725, *p*<.001, *d* = 1.31, CI_95%_ [0.95,1.67]	*t*(52) = 8.796, *p*<.001, *d* = 1.196, CI_95%_ [0.83,1.55]	*F*(3, 153) = 54.000, *p*<.001, η²_*p*_ = .505
BEAQ	49.61 (11.38)	42.68 (9.08), *N* = 56	43.63 (10.75), *N* = 54	42.60 (9.34), *N* = 52	*t*(55) =51.787, *p*<.001, *d* = 0.773, CI_95%_ [0.47,1.07]	*t*(53) = 4.979 *p*<.001, *d* = 0.678, CI_95%_ [0.38,0.97]	*t*(51) = 5.488, *p*<.001, *d* = 0.761, CI_95%_ [0.45,1.07]	*F*(3, 150) = 17.692, *p*<.001, η²_*p*_ = .261
GSES	24.95 (5.02)	29.04 (4.76), *N* = 56	28.73 (5.67), *N* = 55	28.73 (5.38), *N* = 52	*t*(55) = 8.030, *p*<.001, *d* = –1.073, CI_95%_ [–1.40, –0.74]	*t*(53) = 6.263, *p*<.001, *d* = –0.852, CI_95%_ [–1.16, –0.54]	*t(*51) = 7.413, *p*<.001, *d* = –1.028, CI_95%_ [–1.36, –0.69]	*F*(3, 150) = 27.029, *p*<.001, η²_*p*_ = .351
RSE	25.50 (2.98)	24.93 (2.19), *N* = 56	25.28 (2.34), *N* = 54	24.83 (2.79), *N* = 52	*t*(55) = 1.611, *p* = .056, *d* = 0.215, CI_95%_ [0.05,0.48]	*t*(53) = 0.568, *p* = .286, *d* = 0.077, CI_95%_ [0.19,0.34]	*t*(51) = 1.598, *p* = .058, *d* = 0.222, CI_95%_ [0.05,0.50]	*F*(3, 150) = 1582, *p* = .196, η²_*p*_ = .031

Note. FU = follow-up; Y-BOCS = Yale-Brown Obsessive Compulsive Scale; GAF = Global Assessment of Functioning; PHQ-9 = Patient-Health-Questionnaire-9; OCI-R = Obsessive-Compulsive Inventory-Revised; BEAQ = Experiential Avoidance; GSES = General Self-Efficacy Scale; RSE = Rosenberg Self-Esteem Scale; WHOQOL-BREF = global item of the World Health Organization Quality of Life Questionnaire.

#### Prediction of treatment effects.

A linear regression analysis was conducted to examine predictors of OCD symptom severity at the 12-month follow-up. The overall regression model predicting change in OCD symptom severity at the 12-month follow-up was not statistically significant (*p* = .45) and explained 19% of the variance (*R*² = .19). This indicates limited explanatory value of the included predictors. None of the individual predictors reached statistical significance (all *p*’s > .13; see Table 4).

**Table 4:** Regression Analysis with Change in Y-BOCS from Baseline to 12-Month Follow-Up as Outcome Measure, *N* = 54.

Predictor	*β*	*B*	*t*	*p*
Y-BOCS baseline score	0.999	−0.023	−0.071	.943
PHQ-9 baseline score	2.233	0.139	0.596	.554
Gender (male = 0, female = 1^a^)	3.224	−0.794	−0.380	.706
Age	0.999	−0.133	−1.521	.136
Highest education^b^	2.233	1.230	1.371	.177
Early or late onset (early = 1, late = 0)	3.224	2.082	0.951	.347
Comorbidity (yes = 1, no = 0)	0.999	−1.688	−0.071	.446
OCD content, relevant to:				
Contamination (yes = 1, no = 0)	.070	0.999	0.459	.648
Checking (yes = 1, no = 0)	.155	2.233	1.028	.310
Taboo themes (yes = 1, no = 0)	.225	3.224	1.447	.155

Note. Y-BOCS = Yale-Brown Obsessive Compulsive Scale; PHQ-9 = Patient Health Questionnaire-9; ^a^ the option “diverse” was not selected by any participant; ^b^ Secondary school diploma = 1; Upper secondary diploma = 2; University entrance qualification (= A-levels) = 3; Bachelor’s degree = 4; Master’s degree = 5.

To examine predictors of remission status at the 12-month follow-up, all predefined predictors were entered simultaneously into a logistic regression model. The overall logistic regression model predicting remission at the 12-month follow-up was not statistically significant, χ²(10) = 17.37, *p* = .067, explaining a moderate proportion of the variance (Nagelkerke *R*² = .367). Overall, baseline demographic and clinical variables showed limited explanatory value for remission status; lower baseline Y-BOCS scores were significantly associated with a higher likelihood of remission at the 12-month follow-up. At trend level, younger age and early onset was also associated with being in remission at the 12-month follow-up. None of the other predictors were significantly associated with remission at the 12-month follow-up (see Table 5).

**Table 5:** Logistic Regression Analysis with Remission Status (0 = Not in Remission, 1 = in Remission) at 12-Month Follow-Up as Outcome Measure, *N* = 54.

Predictor	*B*	*Wald*	*p*	*Odds Ratio*
Y-BOCS baseline score	−0.244	3.907	.048	0.576
PHQ-9 baseline score	0.041	0.273	.601	0.551
Gender (male = 0, female = 1^a^)	−0.944	1.894	.169	0.325
Age	−0.049	2.719	.099	0.576
Highest education^b^	0.007	0.001	.980	0.551
Early or late onset (early = 1, late = 0)	1.384	3.329	.068	0.325
Comorbidity (yes = 1, no = 0)	−0.292	0.159	.690	0.576
OCD content, relevant to				
Contamination (yes = 1, no = 0)	0.397	0.312	.576	1.488
Checking (yes = 1, no = 0)	0.430	0.355	.551	1.537
Taboo themes (yes = 1, no = 0)	0.729	0.969	.325	2.074

Note. Y-BOCS = Yale-Brown Obsessive Compulsive Scale; PHQ-9 = Patient Health Questionnaire-9; ^a^ the option “diverse” was not selected by any participant; ^b^ Secondary school diploma = 1; Upper secondary diploma = 2; University entrance qualification (= A-levels) = 3; Bachelor’s degree = 4; Master’s degree = 5.

#### Response, remission, and deterioration rates.

From baseline to the 12-month follow up, the mean response rate was 46.28%. Of 55 patients assessed 12 months after treatment, 38 patients (69.1%) qualified as responders (reduction of at least 35% in the total Y-BOCS score) and 28 patients (50.9%) were in remission (score of 12 or below on the Y-BOCS). All patients in remission also qualified as responders. According to the RCI, no clinically relevant deterioration (RCI > 1.96) occurred; however, *n* = 43 (78.2%) showed a reliable improvement (RCI <−1.96).

#### Treatment acceptability.

No patient dropped out during the B4DT treatment. Treatment satisfaction 12 months after treatment as measured with the CSQ–8 was high (total score: *M* = 26.39, *SD* = 6.54). For example, 98.1% evaluated “the quality of the treatment” as good or excellent and affirmed that they “received the help they needed.” In total, 96.2% “would recommend the treatment to a friend with similar problems” (ratings either “yes, definitely” or “yes, I think so”).

## Discussion

### Aim and main findings

The primary aim of the present study was to examine the long-term stability of treatment effects of brief, cERP (B4DT) over a 12-month follow-up period in a German day-patient treatment setting, thereby addressing the lack of long-term data for this therapy format outside of Norway. The results demonstrate that the substantial symptom reductions observed at post-treatment and short-term follow-up were maintained one year after treatment. Importantly, these sustained effects extended beyond clinician-rated OCD symptom severity (Y-BOCS) to multiple secondary outcomes, including psychosocial functioning, self-efficacy, experiential avoidance, quality of life, and uptake of inpatient treatment. Taken together, these findings indicate that cERP yields durable benefits in routine care, underscoring its relevance beyond highly specialized treatment settings.

#### Long-term effectiveness and clinical relevance.

Patients showed large and stable reductions in OCD symptoms, with no evidence of clinically relevant symptom deterioration according to the RCI. At the 12-month follow-up, nearly 70% of the patients met criteria for treatment response and more than half were in remission, with all remitters also qualifying as responders (i.e., reduction of at least 35% in the total Y-BOCS score). These outcomes compare favorably with results reported at post-assessment for ERP [50], suggesting that ERP therapy provided in a concentrated form (cERP) works just as well. Thus, results from our study align with previous findings from Norwegian cohorts [21], despite differences in health care systems and service delivery models.

Beyond symptom severity, sustained improvements were observed across psychosocial functioning and quality-of-life indicators. From a clinical and public health perspective, these broader functional gains are particularly relevant as the burden of OCD is largely driven by long-term impairment in social and occupational domains rather than symptom severity alone. The absence of deterioration further supports the safety and durability of the B4DT approach, suggesting that there is no greater risk associated with delivering ERP in a concentrated form than in the classic format over multiple weeks.

#### Predictors and robustness of outcomes.

Regression analyses revealed no robust demographic or clinical predictors of OCD severity or remission status at the 12-month follow-up except that lower baseline symptom severity was associated with a higher likelihood of remission. Variables such as age, sex, comorbidity, education level, age of onset, and OCD symptom content did not significantly predict long-term outcome.

The lack of strong predictors suggests that once patients successfully complete B4DT, long-term outcomes may be relatively independent of baseline characteristics. This robustness is clinically important as it implies that cERP may be applicable to a broad range of patients and does not require highly selective inclusion criteria—an important consideration for scalable implementation in routine mental health services.

#### Maintenance of gains and follow-up treatment utilization.

A central finding with direct relevance for health systems is the low uptake of inpatient and day-patient services during the 12-month follow-up period. However, given the absence of a control group, direct comparisons to expected service utilization under treatment-as-usual conditions are not possible, and any comparisons should be interpreted with caution. In addition, the potential contribution of the 3-month booster session to the maintenance of gains was not examined and cannot be disentangled from the overall treatment effects. In the present study, the booster session was systematically offered to all participants as part of the treatment program. However, prior research suggests that B4DT outcomes remain largely stable even when excluding patients receiving additional follow-up treatment, indicating that sustained effects are not solely attributable to this one session [28].

To contextualize these findings, they can be considered alongside data from specialized inpatient care in Germany. Intensive inpatient programs with a strong focus on exposure and response prevention (ERP), including therapist-accompanied exposures, are available only in a limited number of specialized centers. These programs typically report outcomes from baseline to discharge rather than longer-term follow-up. In a large sample of 1,596 inpatients with OCD, Herzog et al. [51] reported substantial symptom reductions following specialized inpatient treatment (*g* = 1.34; mean length of stay = 54.9 days).

Importantly, these estimates reflect short-term treatment effects at discharge and are therefore not directly comparable with the 12-month follow-up outcomes reported in the present study. Nevertheless, they provide a useful reference point, suggesting that the magnitude of symptom change observed here is within a similar range despite differences in study design, patient populations, treatment intensity, and assessment time frames.

Within these limitations, the comparatively low reliance on subsequent inpatient or day-patient services observed after B4DT may suggest that clinical improvements were maintained without extensive use of additional specialized care. However, this interpretation remains tentative and cannot be causally attributed to the intervention.

#### Acceptability and implementation.

B4DT demonstrated excellent acceptability, with no dropouts during the treatment week, very high treatment satisfaction at the 12-month follow-up, and follow-up retention exceeding 94%. Such levels of adherence and retention are notable given the intensity of the intervention and contrast with the dropout rates commonly reported for standard ERP in routine outpatient care [18]. Given the substantial clinical and economic costs associated with treatment dropout, including wasted resources and poorer outcomes, the exceptionally low dropout rates observed for B4DT may represent an important mechanism underlying its effectiveness and scalability [52].

High acceptability (e.g., explicit treatment recommendation) is a key prerequisite for dissemination and implementation. The present findings indicate that cERP can be feasibly delivered within a specialized medical university-based day-patient treatment setting and is well tolerated by patients, supporting its integration into existing mental health service structures.

#### Health-economic and public health implications.

Given the high prevalence, chronicity, and economic burden of OCD [3,9,11], interventions that combine strong effectiveness with efficient use of therapeutic resources are of substantial public health importance. B4DT may therefore represent a resource-efficient alternative or complement to standard outpatient care, with implications for reducing treatment completion time, optimizing service capacity, and improving access to evidence-based treatment.

#### Conceptual interpretation and future research.

Although the present study was not designed to examine mechanisms of change, the long-term stability of the outcomes is compatible with models emphasizing skill acquisition, increased functional capacity, and sustained behavioral change rather than short-term symptom reduction alone. However, these interpretations remain speculative and should be tested directly in future studies.

Further studies should integrate long-term follow-up assessments with detailed process measures and formal health-economic evaluations. Randomized controlled trials comparing cERP with standard care models across different health systems will be essential to further clarify its comparative effectiveness, cost-effectiveness, and implementation potential.

### Limitations

Several limitations should be acknowledged. First, the uncontrolled design limits causal conclusions regarding the superiority of B4DT relative to other treatment approaches. Second, the moderate sample size may have limited statistical power for detecting predictors of long-term outcome. Third, several secondary outcomes relied on self-report measures, which may be subject to response biases. Despite these limitations, the high retention rate and consistent pattern of findings across outcomes strengthen confidence in the results.

## Conclusion

This study provides novel evidence that B4DT, a brief, cERP intervention, can achieve substantial and durable improvements in obsessive–compulsive disorder outcomes over a 12-month period when implemented outside its original Scandinavian context. By demonstrating sustained symptom reduction, functional recovery, and high acceptability in a German day-patient treatment setting, the findings extend existing evidence beyond highly specialized cERP environments.

From a global mental health perspective, this work adds important insight into how evidence-based psychotherapy can be delivered in a more efficient, sustainable, and scalable manner. The observation that long-term clinical benefits were largely maintained with minimal reliance on subsequent inpatient or day-patient treatment is particularly relevant for health systems facing limited specialist capacity and growing demand for mental health services. These findings suggest that durable outcomes following brief, concentrated interventions do not necessarily depend on continued access to resource-intensive care.

By addressing both clinical effectiveness and service utilization, this study contributes to a more comprehensive understanding of the real-world sustainability of brief psychotherapy models. The results underscore the potential of concentrated treatment formats to reduce treatment gaps, optimize use of scarce mental health resources, and improve access to effective care across diverse health care systems. As such, cERP represents a promising strategy for strengthening mental health service delivery and advancing global efforts to address the long-term burden of OCD.

## Supporting information

S1 Table TREND Statement Checklist. (PDF)

S2 File Trial Study Protocol. (PDF)

S3 File B4DT_Trail_Protocol_Original_jelinek. (DOCX)
