# Supplementary material for: Brief intervention, lasting impact: One-year outcomes of the Bergen 4-day treatment for OCD in Germany
Source: PLoS One. 2026 Jun 25;21(6):e0350615. doi: 10.1371/journal.pone.0350615 (PMC13298903; doi:10.1371/journal.pone.0350615)
Supplement: S2 File — (PDF) [file pone.0350615.s002.pdf]

Trial Study Protocol

The study protocol was submitted to and approved by the Local Psychological Ethics Committee at the Center for Psychosocial Medicine (LPEK), University Medical Center Hamburg-Eppendorf, Germany, prior to the start of the trial (approval date: July 6, 2022).

Translated verison (Orignal in German)

Title:
The Bergen 4-Day Treatment for Obsessive-Compulsive Disorder: A Pilot Study

Principal Investigator:
Prof. Dr. Lena Jelinek
Martinistraße 52
20246 Hamburg, Germany

Background

The aim of the present study is to evaluate the Bergen 4-Day Treatment (B4DT) in patients with obsessive-compulsive disorder (OCD) within a day clinic setting using a monocentric, single-arm pilot design.

In the B4DT format, patients diagnosed with OCD receive individually tailored concentrated exposure therapy over four consecutive days, supported by a team of therapists. The treatment is delivered in a group setting with a 1:1 therapist-to-patient ratio.

Preliminary studies from Norway have demonstrated that more than 90% of patients respond to B4DT (defined as a reduction >35% on the Yale-Brown Obsessive Compulsive Scale, Y-BOCS), and that 70% were classified as in remission (≤12 points on the Y-BOCS) 1–4 years after treatment (e.g., Hansen et al., 2018, 2019).

Recently, the first randomized controlled trial (RCT) of B4DT was completed, comparing the intervention to a self-help condition and a waitlist control (Launes et al., 2019). Results showed that 93.8% of patients in the B4DT condition responded to treatment, compared to 12.5% in the self-help condition. At 3-month follow-up, 69% of patients were in remission and 31% had improved.

To date, B4DT had not been implemented in Germany.

Objective

The proposed study aims to investigate the feasibility and effectiveness of B4DT for OCD in the day clinic of the Department for Anxiety and Obsessive-Compulsive Disorders at the University Medical Center Hamburg-Eppendorf.

The primary outcome is the improvement in OCD symptom severity across the 4-day treatment period and at follow-up assessments at 3 and 12 months.

Our hypothesis is that treatment with B4DT over a 4-day intervention period will result in a significant reduction in OCD symptom severity from baseline (t0) to post-treatment (t1), as well as sustained improvement at 3-month (t2) and 12-month (t3) follow-up.

Methods

Patients will be recruited from the day clinic for anxiety and obsessive-compulsive disorders at the University Medical Center Hamburg-Eppendorf. Following two pilot groups of three patients each, a total of N = 25 partial inpatient patients with OCD will be enrolled. The sample size is based on a comparable pilot study conducted in Iceland (N = 19; Davíðsdóttir et al., 2019).

Therapists will be trained by the Norwegian developers of the treatment format.

To ensure standardized informed consent, patients will receive a detailed written description of the study procedures prior to treatment.

At baseline (t0), patients will be assessed using the MINI diagnostic interview and the Yale-Brown Obsessive Compulsive Scale (Y-BOCS; primary outcome measure). Additionally, patients will complete questionnaires assessing other psychopathological symptoms (secondary outcomes, e.g., anxiety, depression, quality of life).

After treatment (t1), primary and secondary outcomes will be reassessed, along with patients' subjective evaluation of the B4DT.

Follow-up assessments will be conducted 3 months (t2) and 12 months (t3) after the intervention.

Expected Benefit

The proposed study contributes to the evaluation of a novel, concentrated treatment format for obsessive-compulsive disorder. This intervention has the potential to significantly reduce symptom burden within a short time frame while maintaining long-term effects, thereby contributing to the improvement of OCD treatment in Germany.

References

Davíðsdóttir, S. D. et al. Implementation of the Bergen 4-Day Treatment for Obsessive Compulsive Disorder in Iceland. *Clinical Neuropsychiatry*, 16, 33–38 (2019).

Hansen, B., Hagen, K., Öst, L.-G., Solem, S. & Kvale, G. The Bergen 4-Day OCD Treatment Delivered in a Group Setting: 12-Month Follow-Up. *Frontiers in Psychology*, 9 (2018).

Hansen, B., Kvale, G., Hagen, K., Havnen, A. & Öst, L.-G. The Bergen 4-Day Treatment for OCD: Four-Year Follow-Up of Concentrated ERP in a Clinical Mental Health Setting. *Cognitive Behaviour Therapy*, 48, 89–105 (2019).

Kvale, G. et al. Successfully treating 90 patients with obsessive compulsive disorder in eight days: The Bergen 4-Day Treatment. *BMC Psychiatry*, 18, 323 (2018).

Launes, G. et al. The Bergen 4-Day Treatment for Obsessive-Compulsive Disorder: Does It Work in a New Clinical Setting? *Frontiers in Psychology*, 10 (2019).
