# Supplementary material for: Brief intervention, lasting impact: One-year outcomes of the Bergen 4-day treatment for OCD in Germany
Source: PLoS One. 2026 Jun 25;21(6):e0350615. doi: 10.1371/journal.pone.0350615 (PMC13298903; doi:10.1371/journal.pone.0350615)
Supplement: S3 File — (DOCX) [file pone.0350615.s003.docx]

# Trial Study Protocol

The study protocol was submitted to and approved by the Local Psychological Ethics Committee at the Center for Psychosocial Medicine (LPEK), University Medical Center Hamburg-Eppendorf, Germany before the trial began (approval date 06 July 2022).

Original (German)

Titel: Bergen 4-Day Treatment bei Zwangsstörung: eine Pilotstudie

Studienleiterin: Prof. Dr. Lena Jelinek
Martinistraße 52
20246 Hamburg

Hintergrund:

Das Ziel der vorliegenden Studie ist die Evaluation der Wirksamkeit des Bergen 4-Day Treatment (B4DT) bei Patient:innen mit Zwangsstörung im tagesklinischen Setting anhand einer monozentrischen, einarmigen Pilotstudie. Im B4DT erhalten Patient:innen mit der Diagnose einer Zwangsstörung an vier aufeinanderfolgenden Tagen eine individuell zugeschnittene und von einem Therapeut:innenteam unterstützte Expositionstherapie. Diese wird in einem Gruppensetting durchgeführt, in dem das Verhältnis zwischen Patient:innen und Therapeut:innen 1:1 beträgt. Vorarbeiten aus Norwegen konnten zeigen, dass mehr als 90% der Patient:innen auf das B4DT ansprechen (Reduktion > 35% auf der Y-BOCS) und 70% 1-4 Jahre nach der Teilnahme an der B4DT als remittiert (≤ 12 Punkte auf der Y-BOCS) eingestuft werden konnten (zB. Hansen et al., 2018, 2019). Kürzlich wurde eine erste randomisierte kontrollierte Studie (RCT) der B4DT abgeschlossen, in der sie mit einer Selbsthilfeintervention und einer Wartebedingung verglichen wurde (Launes et al., 2019). Die Ergebnisse zeigten, dass 93.8% auf die Behandlung in der B4DT-Bedingung ansprachen. Bei den Patient:innen in der Selbsthilfeintervention sprachen 12.5% auf die Behandlung an. Bei der 3-monatigen Nachuntersuchung waren 69% der Patient:innen remittiert und 31% hatten sich gebessert. Bisher wurde das B4DT in Deutschland nicht angewendet.

Ziel: Die beantragte Studie beabsichtigt die Machbarkeit und Wirksamkeit des B4DT bei Zwangsstörung in der Tagesklinik der Station für Angst- und Zwangserkrankungen zu untersuchen. Hauptzielparameter ist die Verbesserung der Zwangssymptomatik über den Behandlungszeitraum von 4 Tagen bzw. über den Follow-up Zeitraum von 3 und 12 Monaten.

Unsere Hypothese lautet: Die Behandlung über einen Interventionszeitraum von 4 Tagen mit B4DT bewirkt eine Verbesserung der Zwangssymptomatik von Beginn (t0) und zum Ende der Therapie (t1) sowie eine nachhaltige Besserung nach 3 Monaten (t2) und 12 Monaten (t3).

Vorgehen: Über die Tagesklinik für Angst- und Zwangsstörungen des Universitätsklinikum Hamburg-Eppendorf sollen nach zwei Pilotdurchgängen à 3 Patient:innen, N = 25 teilstationäre Patient:innen mit einer Zwangsstörung rekrutiert werden. Die Stichprobengröße ist an eine vergleichbare Pilotstudie in Island angelehnt (N = 19, Davíðsdóttir et al., 2019). Die Therapeut:innen werden von den norwegischen Entwicklern des Behandlungsformats geschult. Für eine standardisierte Aufklärung erhalten die Patient:innen vor der Behandlung eine detaillierte Beschreibung über den Studienablauf. Vor der Behandlung (t0) werden die Patient:innen mit dem MINI Interview und der Y-BOCS (primärer Outcome) gescreent und füllen weitere Fragebögen zu anderen psychopathologischen Symptomen (sekundäre Outcomes, z.B. Ängste, Depression, Lebensqualität) aus. Nach der Behandlung werden die primären und sekundären Outcomes erneut erhoben sowie die subjektive Bewertung des B4DT (t1). Drei (t2) und 12 (t3) Monate nach der Intervention werden die Outcomes erneut erhoben.

Erwarteter Nutzen: Die beantragte Studie leistet einen Beitrag zur Evaluation eines neuen, konzentrierten Therapieformats für Zwangsstörung. Dieses birgt das Potential die Symptombelastung innerhalb kurzer Zeit sowie nachhaltig signifikant zu reduzieren und kann damit beitragen die Behandlung von Zwangsstörungen in Deutschland zu verbessern.

Literatur

Davíðsdóttir, S. D. *et al.* Implementation of the Bergen 4-Day Treatment for Obsessive Compulsive Disorder in Iceland. *Clin. neuropsychiatry* **16**, 33–38 (2019).

Hansen, B., Hagen, K., Öst, L.-G., Solem, S. & Kvale, G. The Bergen 4-Day OCD Treatment Delivered in a Group Setting: 12-Month Follow-Up. *Frontiers in Psychology*  vol. 9 (2018).

Hansen, B., Kvale, G., Hagen, K., Havnen, A. & Öst, L.-G. The Bergen 4-day treatment for OCD: four years follow-up of concentrated ERP in a clinical mental health setting. *Cogn. Behav. Ther.* **48**, 89–105 (2019).

Kvale, G. *et al.* Successfully treating 90 patients with obsessive compulsive disorder in eight days: the Bergen 4-day treatment. *BMC Psychiatry* **18**, 323 (2018).

Launes, G. *et al.* The Bergen 4-Day Treatment for Obsessive-Compulsive Disorder: Does It Work in a New Clinical Setting? *Frontiers in Psychology*  vol. 10 (2019).
